# Glandular trichomes of *Robinia viscosa* Vent. var. *hartwigii* (Koehne) Ashe (Faboideae, Fabaceae)—morphology, histochemistry and ultrastructure

**DOI:** 10.1007/s00425-020-03513-z

**Published:** 2020-11-12

**Authors:** Agata Konarska, Barbara Łotocka

**Affiliations:** 1 Department of Botany and Plant Physiology, University of Life Sciences in Lublin, Akademicka 15, 20-950 Lublin, Poland; 2 Department of Botany, Warsaw, University of Life Sciences, Nowoursynowska 159, 02-776 Warsaw, Poland

**Keywords:** Leguminosae, Secretory hair, Secondary metabolites, Alkaloids and tannins, Sticky secretion

## Abstract

**Main Conclusion:**

Permanent glandular trichomes of *Robinia viscosa* var. *hartwigii* produce viscous secretion containing several secondary metabolites, as lipids, mucilage, flavonoids, proteins and alkaloids.

**Abstract:**

*Robinia viscosa* var. *hartwigii* (Hartweg’s locust) is an ornamental tree with high apicultural value. It can be planted in urban greenery and in degraded areas. The shoots, leaves, and inflorescences of this plant are equipped with numerous persistent glandular trichomes producing sticky secretion. The distribution, origin, development, morphology, anatomy, and ultrastructure of glandular trichomes of Hartweg's locust flowers as well as the localisation and composition of their secretory products were investigated for the first time. To this end, light, scanning, and transmission electron microscopy combined with histochemical and fluorescence techniques were used. The massive glandular trichomes differing in the distribution, length, and stage of development were built of a multicellular and multiseriate stalk and a multicellular head. The secretory cells in the stalk and head had large nuclei with nucleoli, numerous chloroplasts with thylakoids and starch grains, mitochondria, endoplasmic reticulum profiles, Golgi apparatus, vesicles, and multivesicular bodies. Many vacuoles contained phenolic compounds dissolved or forming various condensed deposits. The secretion components were transported through symplast elements, and the granulocrine and eccrine modes of nectar secretion were observed. The secretion was accumulated in the subcuticular space at the trichome apex and released through a pore in the cuticle. Histochemical and fluorescence assays showed that the trichomes and secretion contained lipophilic and polyphenol compounds, polysaccharides, proteins, and alkaloids. We suggest that these metabolites may serve an important function in protection of plants against biotic stress conditions and may also be a source of phytopharmaceuticals in the future.

## Introduction

Many plants from different botanical families produce diverse external secretory structures, e.g. nectaries, hydathodes, and glandular trichomes releasing secretion with a varying composition.

Glandular trichomes are characteristic for representatives of many botanical families, e.g. Asteraceae (Muravnik et al. 2016; Xiao et al. 2016), Cannabaceae (Mahlberg and Kim 2004), Geraniaceae (Boukhris et al. 2013), Lamiaceae (Maurya et al. 2019), Orobanchaceae (Konarska and Chmielewski 2020), Scrophulariaceae (Attar et al. 2007), Solanaceae (Bergau et al. 2016), Verbenaceae (Silva et al. 2016), and Fabaceae (Barros et al. 2017a; Vargas et al. 2019).

Glandular trichomes produce, store, or secrete mainly lipophilic substances, i.e. fats, waxes, essential oils, and resins (Stojičić et al. 2016; Citti et al. 2019; Konarska and Chmielewski 2020). However, the secretion often contains polysaccharides and proteins (Tozin and Rodrigues 2017), alkaloids (Munien et al. 2015), or phenolic compounds (Jachuła et al. 2018). It has been found that trichome secretion deters herbivores and acts as a natural pesticide (LoPresti 2015; Murungi et al. 2016), inhibits the growth of fungal and bacterial pathogens (Steiner et al. 2015; Rodriguez et al. 2018), acts as a food or signalling attractant (Werker 2000; Płachno et al. 2019), contributes to the spread of fruits (Heinrich et al. 2002), and protects against atmospheric oxidative stress (Li et al. 2018). Many of these specialised metabolites are used as pharmaceuticals, nutraceuticals, cosmetic components, and food additives, e.g. flavours and fragrances (Duke et al. 2000; Aharoni et al. 2006; Tissier 2012).

As emphasised by many researchers, the distribution and structure of glandular trichomes and the secondary metabolites contained in their secretion help to determine and elucidate the function of these structures. They also serve as important diagnostic traits in taxonomy and systematics for assessment of relatedness between taxa (Fortuna-Perez et al. 2012; Meira et al. 2014; Muravnik et al. 2019). Additionally, investigations of glandular trichomes and their secretion may contribute greatly to elucidation of the ecology and evolution of the genus (Eiji and Salmaki 2016; Khosroshahi Eyvazadeh and Salmaki 2019) and identification and standardisation of medicinal raw materials (Karlygash et al. 2016; Livingston et al. 2019).

Nevertheless, two general types of trichomes are distinguished: capitate and peltate trichomes (Evert 2006; Maffei 2010). The morphology of secretory trichomes may vary highly both between and within families and genera. Secretory trichomes are formed via divisions of epidermis cells. The cells of secretory trichomes usually have dense cytoplasm and numerous mitochondria. Additionally, plastids, Golgi apparatus, and endoplasmic reticulum may be present in the cells of these structures, depending on the composition of the secretion (Fahn 1988; Evert 2006).

Various morphotypes of glandular trichomes have been distinguished in the Fabaceae family, e.g. in the subfamilies Cercidoideae (Marinho et al. 2015), Caesalpinioideae (Coutinho et al. 2015; Silva et al. 2017), and Faboideae (Matos and Paiva 2012; Flores et al. 2019; Vargas et al. 2019). Representatives of the family Fabaceae often have other exogenous secretory structures, such as floral nectaries (Konarska 2020), extrafloral nectaries (Gonzalez and Marazzi 2018), and osmophores (Marinho et al. 2018). Additionally, endogenous secretory structures such as cavities and/or canals have been described (Milani et al. 2012; Mendes et al. 2019).

*Robinia* L. from the tribe Robinieae, subfamily Faboideae (Fabaceae), is native to North America, whereas *Robinia pseudoacacia* L., *R. hispida* L., *R. neomexicana* A. Grey, *R. viscosa* Vent., and their hybrids are grown as ornamental plants (Chan 2012; Oprea et al. 2012) and used for recovery of degraded areas (Boring and Swank 1984) in many European countries. The plants are considered poisonous to animals and humans (Mezzasalma et al. 2017; Stegelmeier and Davis 2018) due to the content of lectins in their organs, especially in the bark and seeds (Rabijns et al. 2001; Hu et al. 2015).

*Robinia viscosa* is an easily recognisable species, as its shoots, inflorescences, and fruits are covered by numerous trichomes producing viscous secretion. The small *R. viscosa* trees or shrubs produce attractive racemose inflorescences consisting of pink flowers with abundant amounts of nectar (Keresztesi 1983; Chan 2012). Two varieties of this species are known: *R. viscosa* var. *viscosa* L. (clammy locust) and *R. viscosa* var. *hartwigii* (Koehne) Ashe (Hartweg's locust). The abundant glandular trichomes with distinct stalks are a characteristic trait of Hartweg's locust, whereas the glandular trichomes in clammy locust do not have stalks and resemble papillae (Isely and Peabody 1984; Peabody 1984). The available literature data indicate that the origin, developmental stages, and microstructure of *R. viscosa* glandular trichomes as well as the composition and role of their secretion are yet unknown.

The aim of the present study was to provide the first comprehensive report on the morphological, anatomical, and ultrastructural features of glandular trichomes in Hartweg's locust, with special emphasis on their origin, development, production and transport of excretion, and the mode of secretion. The results were obtained with the use of various microscopic techniques. Additionally, to identify the possible ecological functions of the trichomes and the potential therapeutic effects of their secretion, the chemical content of trichome secretion/exudates was analysed with histochemical and fluorescence assays. These microscopic techniques facilitate rapid and cost-efficient preliminary assessment of the content of secondary metabolites and preliminary evaluation of the medicinal potential of the plant in search of new pharmaceuticals (Mercadante-Simões et al. 2014; Demarco 2017; Mownika et al. 2020).

## Material and methods

**Fig. 1:**
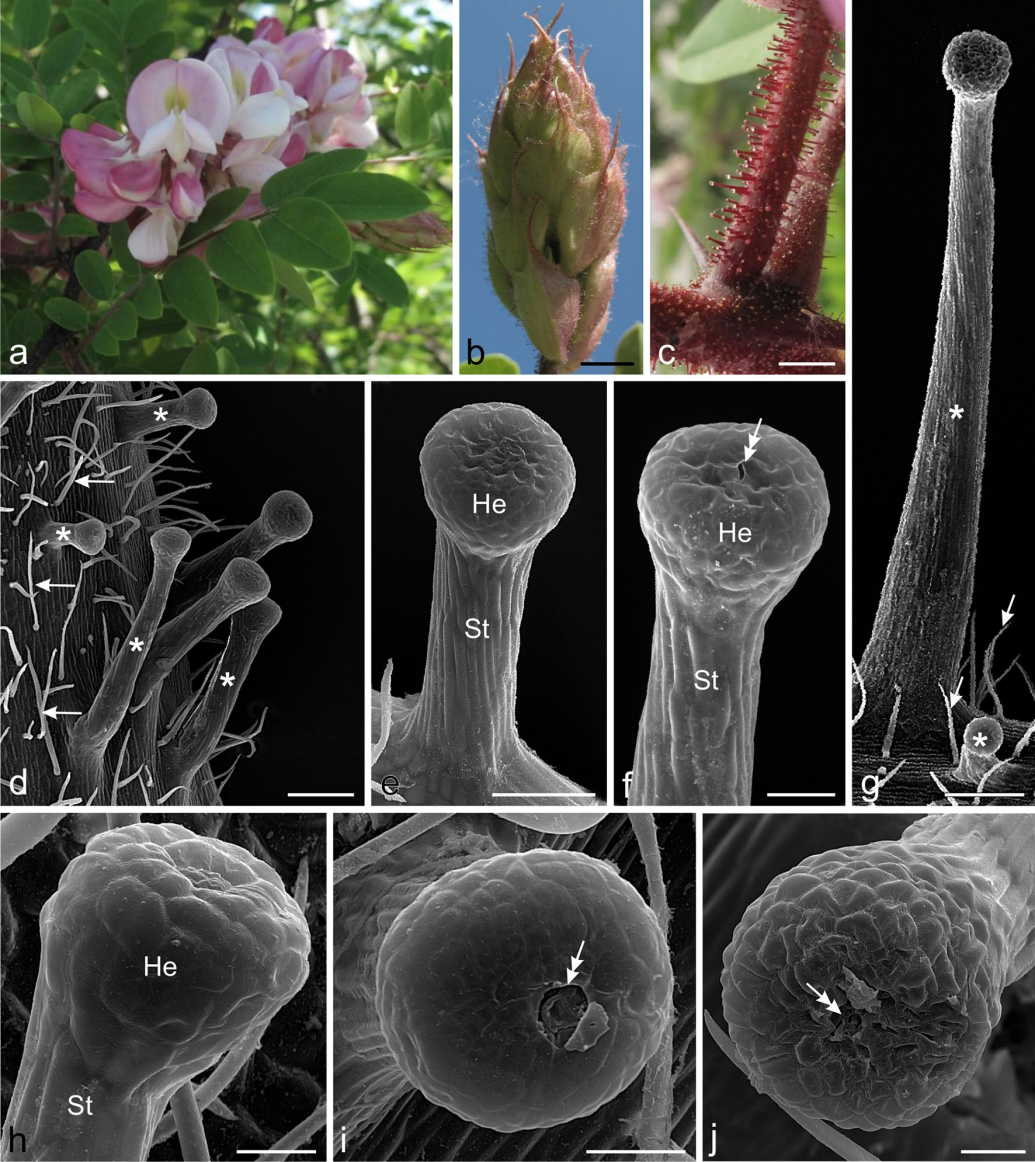
Morphology of the inflorescence and micromorphology of the glandular trichomes of Hartweg's locust. **d–j** SEM. **a** Inflorescence with flowers in different stages of development. **b** Visible inflorescence in the bud stage with bracts covered by glandular trichomes. **c** Peduncles with glandular trichomes. **d** Non-glandular (arrows) and massive glandular (asterisks) trichomes with varied length visible on the pedicle surface. **e–j** Mature glandular trichomes in different stages of activity. **e, f, i, j** Trichomes in the secretory or post-secretory phases with sunken cells (**e, f, j**) and with a pore in the cuticle (double arrows) on the head apex (**f, i, j**). **g** Non-glandular (arrows) and glandular (asterisks) trichomes with substantially varied length visible on the bract surface. **h** Trichome in the pre-secretory phase with convex cell walls of the head; He trichome head, St trichome stalk. Scale bars = 10 mm (**b**), 1 mm (**c**), 200 µm (**d**, **g**), 100 µm (**e**), 50 µm (**f**), 30 µm (**h–j**)

Inflorescences of Hartweg's locust, i.e. a small tree whose shoots, leaves, and flowers bear massive and distinct glandular trichomes, were analysed (Fig. 1a–c). The inflorescences (*n* = 10) were collected randomly in the flowering period in May 2018 and 2019 from the central part of the canopy of five trees growing on a square in Jastrzębowskiego and Rodowicza “Anody” Streets in Warsaw, Poland (52° 09′ 48.0″ N, 21° 02′ 10.9″ E). 2–3 mm long flower buds (*n* = 30) were collected from the apical part of each inflorescence for microscopic examinations. Bracts, middle fragments of flower pedicels, and receptacles (each object *n* = 30) were collected from the middle part of the inflorescences in the full bloom phase. The herbarium specimens used this study were deposited in the Herbarium of the Maria Curie-Sklodowska University in Lublin (LBL P) under number 1001.

Secretory trichomes in various stages of development and activity located on the surface of the examined organs were analysed using light (LM), fluorescence (FM), scanning electron (SEM), and transmission (TEM) electron microscopy and examined with various histochemical and fluorescence assays.

### Scanning electron microscope

Samples of flower pedicels, bracts, and receptacles (each object *n* = 6 from different flowers and trees) were fixed in 4% (*v/v*) glutaraldehyde in 0.1 M phosphate buffer with pH 7.0. Next, the samples were dehydrated in an ethanol series, critical point dried in liquid CO_2_ (Bal-Tec CPD 030 critical point dryer), and coated with gold–palladium with an EMITECH K 550 × sputter coater. Finally, the samples were viewed under a TESCAN/VEGA LMU scanning electron microscope at an accelerating voltage of 30 kV.

### Light microscope

Flower buds, pedicels, bracts, and receptacles (each collected in ten independent randomly selected repeats *n* = 10 from different flowers and trees) were fixed in a mixture of 4% (w/v) paraformaldehyde (Sigma-Aldrich, St. Louis, MO, USA) and 5% (*v/v*) glutaraldehyde (Sigma-Aldrich) in 0.1 M sodium cacodylate buffer (Sigma-Aldrich) pH 7.2 for 6 h at room temperature. Next, they were rinsed four times in cacodylate buffer and post-fixed in osmium tetroxide (0.05%) for 2 h at 4 °C. After washing three times in cacodylate buffer, the specimens were dehydrated in a graded ethanol series (10, 30, 50, and 70% (*v/v*)) two times for 15 min at 4 °C. For better aeration, the samples were stored for several days at 4 °C in a 70% (*v/v*) ethanol solution and finally further dehydrated in a 90%, 96%, and 99.8% ethanol series, substituted with propylene oxide, and infiltrated and embedded in medium-grade of epoxy resin (Sigma-Aldrich; equivalent to Epon 812) following the manufacturer’s formula. The specimens were polymerized at 65 °C for 16 h. The samples were sectioned on a Leica RM2165 microtome (Leica Microsystems), and the Sects. (3 µm thick) were collected on glass slides (Menzel-Gläser, Braunschweig, Germany). For general histology, semi-thin sections were stained with a 1% (*w/v*) aqueous methylene blue-azure II solution (O’Brien and McCully 1981) and examined under an Olympus AX70 ‘Provis’ light microscope (Olympus, Tokyo, Japan) equipped with an Olympus DP50 digital camera (Olympus).

### Transmission electron microscope

Ultrathin Sects. (90 nm thick) for transmission electron microscopy were taken with a Leica UCT ultramicrotome (Leica Microsystems) and stained with a saturated methanolic solution of 0.5% (*w/v*) uranyl acetate (Sigma-Aldrich) followed by incubation in 0.5% (*w/v*) lead citrate (Sigma-Aldrich) (Reynolds 1963). They were examined using an FEI 268D ‘Morgagni’ transmission electron microscope (FEI Company, Hillsboro, OR, USA) equipped with an Olympus-SIS ‘Morada’ digital camera (Olympus). The samples were collected in five independent randomly selected repeats.

### Histochemistry and fluorescence

To determine the distribution and types of secondary metabolites present in the secretory trichomes of Hartweg's locust, hand-cut sections of fresh unfixed and unstained tissue of flower pedicels, bracts, and receptacles (*n* = 5 of each object) were viewed in a 50% aqueous glycerol solution (as a control) and after application of various histochemical assays and fluorochromes (Table 1). Since some histochemistry and fluorescence assays may yield ambiguous results, we used an adequate number of repetitions (*n* = 6) for each technique and independent parallel reaction to eliminate erroneous interpretation. Similar positive results were obtained 4–5 times. The histochemical methods followed standard control procedures suggested by various authors (Table 1 and references therein). Fluorescence was observed with the use of a Cy5 filter set (excitation light: 590–650 nm; barrier filter wavelength: 663–738 nm) and a TRITC filter set (excitation light: 525–565 nm; barrier filter wavelength: 555–600 nm).

**Table 1:** Primary and secondary metabolites identified in the secretory trichomes of *Hartweg’s locust* by histochemical tests and fluorescence assays

Staining	Target compounds	Reference	Colour	Head cells	Stalk cells	Secretion
Sudan IV	Total lipids	Johansen (1940); Lison (1960)	Orange	+	+	+ +
Sudan Red B	Total lipids	Brundrett et al. (1991)	Red	+	+	+ +
Neutral Red under UV	Total lipids	Kirk (1970); Conn (1977)	Blue or green light	–	+ +	+
Auramine O under UV	Lipids	Jensen (1962); Gahan (1984)	White or yellow light	–	+	+
Nile Blue	Acidic lipids (phospholipids, free fatty acids) Neutral lipids (fats, essential oil)	Cain (1947); Jensen (1962)	Blue Pink	+ + –	+ + –	– + +
Nadi reagent	Essential oils	David and Carde (1964)	Violet	–	–	–
Concentrated sulphuric acid	Sesquiterpenes	Geissman and Griffin (1971); Cappelletti et al. (1986)	Yellow/red-brown	–	–	–
Antimony trichloride under UV	Terpenes contain steroids	Hardman and Sofowora (1972)	Blue or yellow light	–	+	+
Ferric chloride	Polyphenols	Johansen (1940)	Dark brown	+ +	+	–
Potassium dichromate	Tannins	Gabe (1968)	Brown	+ +	+ +	–
Vanillin with hydrochloric acid	Tannins	Mace and Howell (1974); Gardner (1975)	Burgundy	+ +	+	–
Aluminium chloride under UV	Flavonoids	Charrière-Ladreix (1976)	Blue or green light	+	+	+
Magnesium acetate under UV	Flavonoids	Charrière-Ladreix (1976)	Blue or green light	+	+	+
UV (autofluorescence)	Phenolic acids Chlorophyll	Griffiths (1958) Buschmann et al. (2000)	Blue light Red light	+ +	+ –	– –
Ruthenium Red	Acidic polysaccharides (mucilage, pectins)	Johansen (1940); Jensen (1962)	Purple	+ +	+	+ +
Periodic acid—Schiff's reagent (PAS)	Neutral polysaccharides (cellulose)	O'Brien and McCully (1981)	Cyclamen	+	+	-
Iodine iodide solution (IKI)	Proteins	Johansen (1940)	Yellow	+	-	+ +
Wagner reagent	Alkaloids	Furr and Mahlberg (1981)	Dark orange	+	-	+ +

+  + intensive, + positive, − negative

Additionally, the diameter of the heads and the total length of mature glandular trichomes (with a clearly developed head and visible secretion in the subcuticular space) from the bracts, receptacles, and flower pedicels (*n* = 50 of each object) were measured. Means and standard deviations (± SD) were calculated for the measured parameters using Excel 7.0 software (Microsoft, Washington, USA). Data were subjected to one-way analysis of variance (ANOVA) and Tukey’s honestly significant difference test for comparison of the means. The analysis was carried out using software STATISTICA 7.0 (StatSoft, Inc., USA). Differences were considered statistically significant at the level of 0.05. The results were documented using a Nikon SE 102 light microscope and a Nikon 90i fluorescence microscope equipped with a digital camera (Nikon Fi1) and NIS-Elements Br 2 software.

## Results

### Distribution and micromorphology of glandular trichomes

The inflorescences of Hartweg's locust were characterised by the presence of glandular trichomes producing viscous secretion (Fig. 1b, c). Trichomes located on the peduncles, pedicels, abaxial surfaces of bracts and sepals, and receptacles exhibited varied distribution and different length values (Fig. 1b–d, g). Particularly large numbers of trichomes were present on the bracts and pedicels, whereas lower abundance was observed on the sepals and receptacles. The heads of the trichomes were located on distinct stalks whose length usually depended on the location of the trichome. The shortest mature glandular trichomes were located on the receptacle surface, while over three-fold longer trichomes were detected on the pedicels (Table 2). The diameter of the heads of the shortest trichomes was on average over two-fold smaller than that in the considerably long trichomes. The differences between the diameter of the trichome heads and the length of the trichomes present on the bracts, receptacles, and pedicles were statistically significant (Table 2). Each organ sampled from the developed inflorescences had both long and short mature trichomes (Fig. 1d, g).

**Table 2:** Characteristics of the trichome length and the diameter of the trichome head of Hartweg’s locust

Part of flower	The means ± SD	
	Trichome length (µm) *n *= 50	Trichome head diameter (µm) *n * = 50
Receptacle	250.1 ± 26.92*	74.1 ± 7.68*
Bract	426.2 ± 42.10*	142.9 ± 18.85*
Pedicle	771.2 ± 141.42*	167.1 ± 10.45*

*Within a column indicate statistically significant differences (= 0.05)

The surface of the glandular trichomes was covered by a smooth cuticle. The cells of the outer layer of the stalk were elongated along its long axis, whereas the surface of the head was formed by isodiametric cells with a polyhedral outline (Fig. 1e, f, h–j). Cells located on the periphery of the head had convex outer peripheral walls in trichomes in the pre-secretory phase (Fig. 1h). The walls were either flat or sunken, especially at the apex of the head in trichomes in the secretory and post-secretory phases (Fig. 1e, f, i, j). The apices of the heads in many trichomes in the secretory and post-secretory phases had small pores in the cuticle, through which the secretion was released (Fig. 1f, i, j). After secretion, the cells of the apical part of the head most often shrank, and their walls wrinkled and collapsed (Fig. 1e, f, j). Numerous much shorter unicellular non-glandular trichomes were visible between the secretory trichomes on the surface of the examined organs (Fig. 1d, g).

### Anatomy of glandular trichomes

**Fig. 2:**
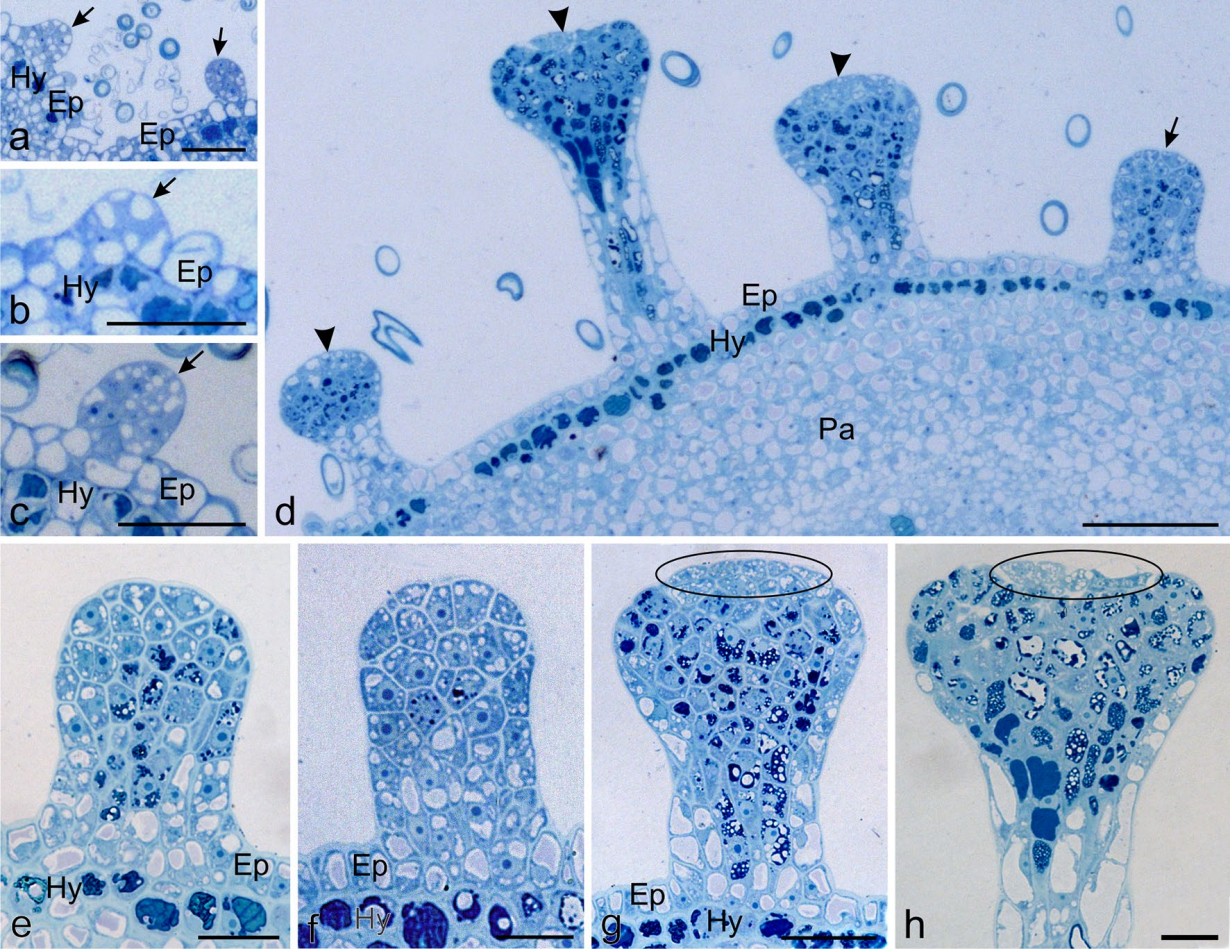
Anatomy of glandular trichomes of Hartweg's locust from the initial to mature stages of development (LM). **a** Cross-section of sepals in a flower bud with trichomes (arrows) in the initial stage of development. **b, c** Immature trichomes (arrows) in the initial stage of development visible on the sepals. **d** Cross-section of pedicles with immature (arrow) and mature (arrowheads) trichomes. **e, f** Immature trichomes from the receptacle (**e**) and pedicle (**f**). Note the dark phenol deposits in some vacuoles. **g, h** Mature trichomes with visible dark phenol deposits in the vacuoles of many head and stalk cells. **g** Trichome in the pre-secretory phase with an oval head. Note the group of cells densely arranged relative to each other and the free phenols deposits (ellipse) on the trichome apex. **h** Trichomes in the secretory or post-secretory phases with flattened heads. Note the group of cells loosely arranged relative to each other and the free phenols deposits (ellipse) on the trichome apex; *Ep* epidermis, *Hy* hypodermis, *Pa* parenchyma. Scale bars = 100 (d), 30 µm (a–c, g, h), 10 µm (e, f)

The glandular trichomes on the examined organs (pedicels, bracts, sepals, and receptacles) of Hartweg's locust were found to undergo not only different phases of activity but also different stages of development. Immature trichomes in the initial development stages were visible mainly on the abaxial surface of the sepals in the bud stage (Figs. 2a–c, 7a–c). The trichomes were formed through mitotic divisions of epidermis cells. Such underdeveloped trichomes originating from cells with visible walls formed in final anticlinal and/or periclinal divisions were also visible on the surface of the other fully developed parts of the inflorescences, i.e. the pedicels, bracts, and receptacles (Fig. 2d–f). During the development of trichomes, anticlinal cell divisions, which led to an increase in the diameter of the head, were initially observed. Next, the length of the stalk increased through periclinal divisions contributing to the elongation of the trichomes. The immature trichomes were built of densely arranged cells forming small capitate protrusions without a clearly visible stalk (Figs. 2c–f, 7a–c). The cells of the youngest trichomes, which were composed of their smallest number, most often had dense cytoplasm and a large cell nucleus (Fig. 2e, f). In the subsequent stages of development, some cells of the young trichomes had large and most often colourless vacuoles; less frequently, they contained intensely coloured precipitates of phenolic compounds (Figs. 2e–f, 7a–c).

**Fig. 3:**
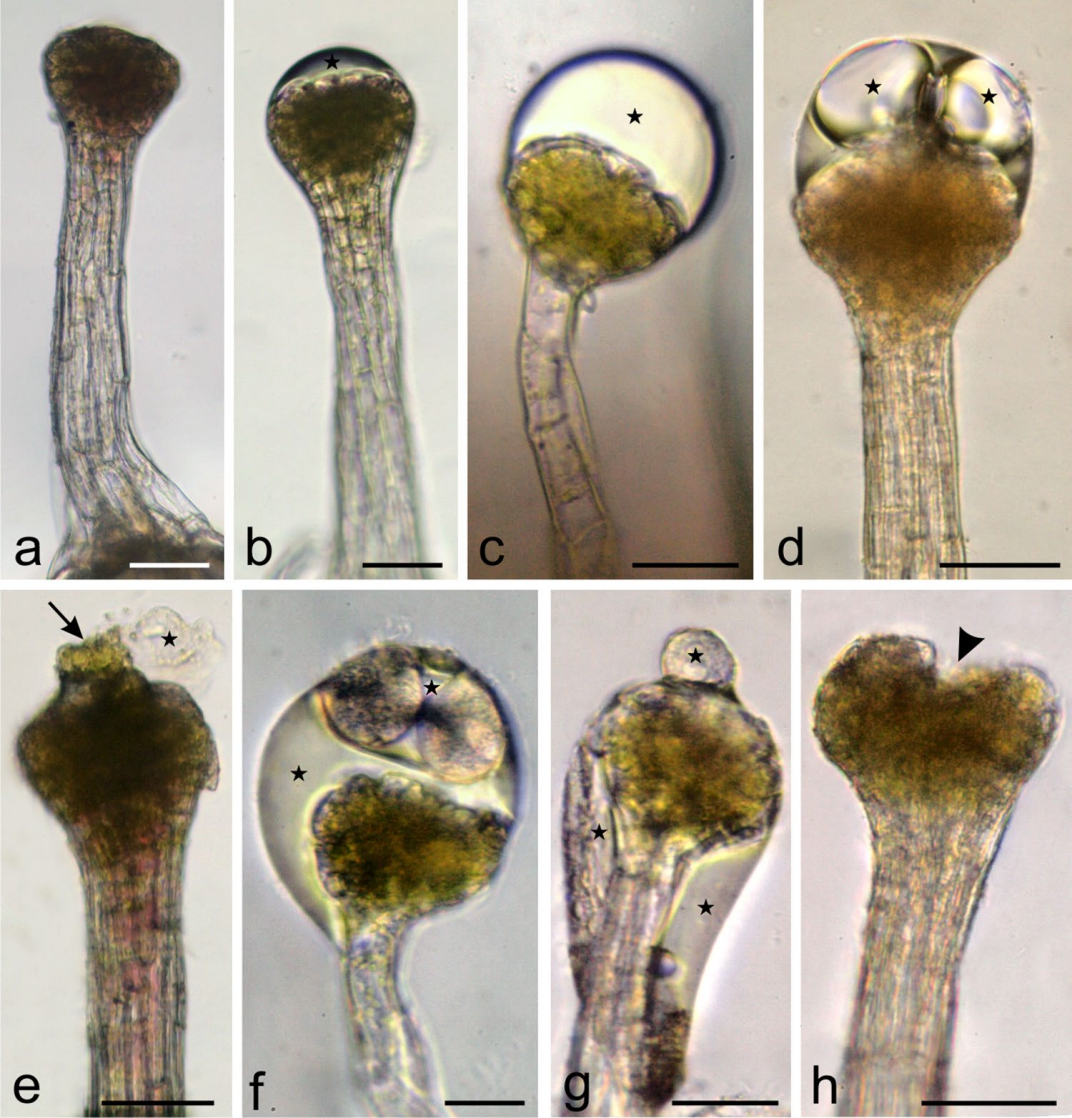
Consecutive **s**tages of the activity of the glandular trichomes of Hartweg's locust observed in fresh samples (LM). **a** Trichome in the pre-secretory phase. **b**–**h** Trichomes in the secretory phase. Visible consecutive stages of accumulation of the secretion in the subcuticular spaces (stars). **e** Trichome with a visible rupture in the cuticle (arrow) and secretion (star). **f, g** Trichomes covered with sticky secretion (stars). **h**Trichome in the post-secretory phase with a depression at the trichome apex (arrowhead). Scale bars = 100 µm (a–h)

The mature secretory trichomes from the pedicels, bracts, and receptacles in the longitudinal semi-thin sections were characterised by a massive multicellular stalk and a clearly formed multicellular head with an evidently flattened shape (Figs. 2d, g, h, 3a–h). The stalk cells were elongated at the long axis of the trichome, thus forming 5–6 vertical rows (bands), whereas the head cells had diverse but close to isodiametric shapes. The vacuoles of cells located on the periphery of the stalk were typically colourless, whereas the vacuoles of cells from the central part of the stalk and close to the head were often filled with dark content (Fig. 2g, h). Vacuoles with similar content were also observed in the cells of the head, especially in its central part (Fig. 2g, h). In turn, the cells of the apical part of the trichome head most often had prominent cell nuclei and were less intensively vacuolated. The vacuoles of this part of the head usually had no tannin deposits (Fig. 2g, h). Depending on the phase of activity of the trichome, the cells of the apical part of the head tightly adhered to each other in oval-headed trichomes undergoing the pre-secretory phase (Fig. 2g) or exhibited looser arrangement in trichomes with cup-like heads in the secretory or post-secretory phases (Fig. 2h). Vacuoles with dark content were also seen in the hypodermis cells of the pedicels, bracts, sepals, and receptacles (Fig. 2a–g).

The fresh mature trichomes viewed under LM had typically a colourless stalk and a green-olive or brown-burgundy head (Fig. 3a–h). The colourless secretion produced in the trichome cells accumulated gradually and filled the subcuticular space (Fig. 3b–d). Next, it was released through a pore in the head apex cuticle (Fig. 3e). Due to its large amount and high viscosity, the secretion often formed a thick layer on the head and stalk surface (Fig. 3f, g). Its high viscosity was responsible for the adherence of small insects, pollen grains, fungal spores, and algal cells to the surface of the trichomes. A distinct concavity at the apices of the trichome heads was visible in the post-secretory phase (Fig. 3h).

### Histochemistry and fluorescence tests

The histochemical assays and fluorescence microscopy observations of the glandular trichomes and their secretions revealed the presence of lipophilic substances (neutral and acidic lipids and steroid-containing terpenes) and hydrophilic compounds such as polyphenols (phenol acids, tannins, and flavonoids), polysaccharides (neutral and acidic), proteins, and alkaloids (Table 1).

**Fig. 4:**
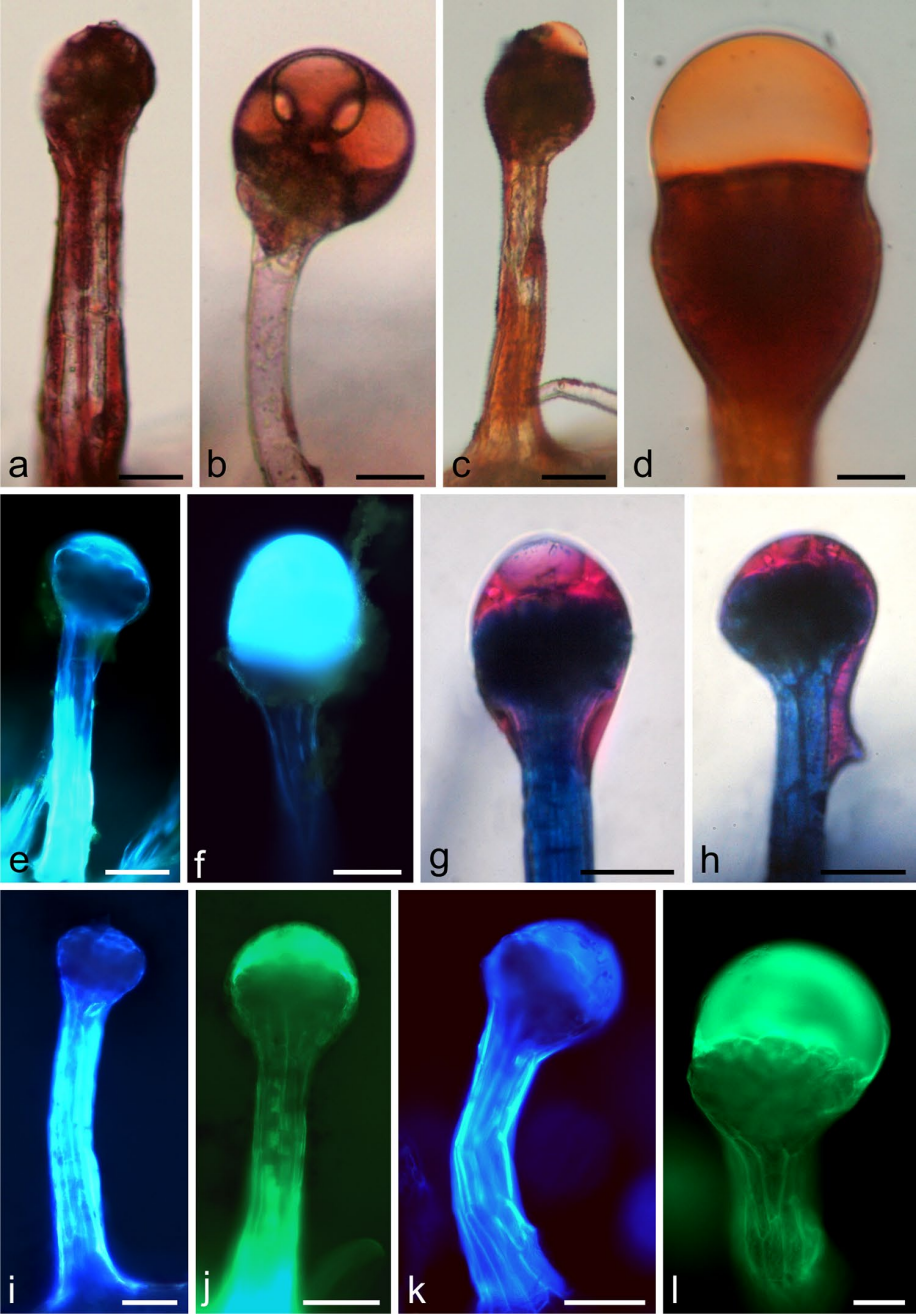
Histochemistry and fluorescence of glandular trichomes of Hartweg's locust* –* lipophilic compounds. **a–d, g, h** – LM. **e, f, i–l**—FM. **a, b** Staining of total lipids with Sudan Red B. **c, d** Staining of total lipids with Sudan IV. **e, f** Fluorescence of lipids with the Auramine O fluorochrome in the Cy 5 filter set. **g, h** Staining of neutral and acidic lipids with Nile Blue. **i, j** Fluorescence of lipids with the Auramine O fluorochrome in the Cy5 (**i**) and TRITC (**j**) filter sets. **k, l** Fluorescence of steroid-containing terpenes with the antimony trichloride fluorochrome in the Cy5 (**k**) and TRITC (**l**) filter sets. Scale bars = 100 µm (a–c, e–k), 50 µm (d, l)

The Sudan Red and Sudan IV reagents revealed the presence of lipids in the stalk and head cells and, in particular, in the trichome secretions (Fig. 4a–d). The presence of lipophilic compounds was also confirmed by the fluorescence of lipids contained in the secretions and stalk cells in the presence of the auramine O (Fig. 4e, f) and Neutral Red (Fig. 4i, j) fluorochromes. As confirmed by the reaction with Nile Blue, the lipophilic compounds contained in the secretion were represented by neutral lipids, whereas the stalk and head cells exhibited the presence of acidic lipids (Fig. 4g, h). In turn, the steroid-containing terpenes present in the outer layers of the trichome stalks and in the secretions emitted intense fluorescence in the reaction with antimony trichloride (Fig. 4k, 1). No sesquiterpenes were detected by the reaction with concentrated sulphuric acid. Similarly, the reaction with the Nadi reagent did not show any content of essentials oils and oleoresins in the trichome cells and secretion.

**Fig. 5:**
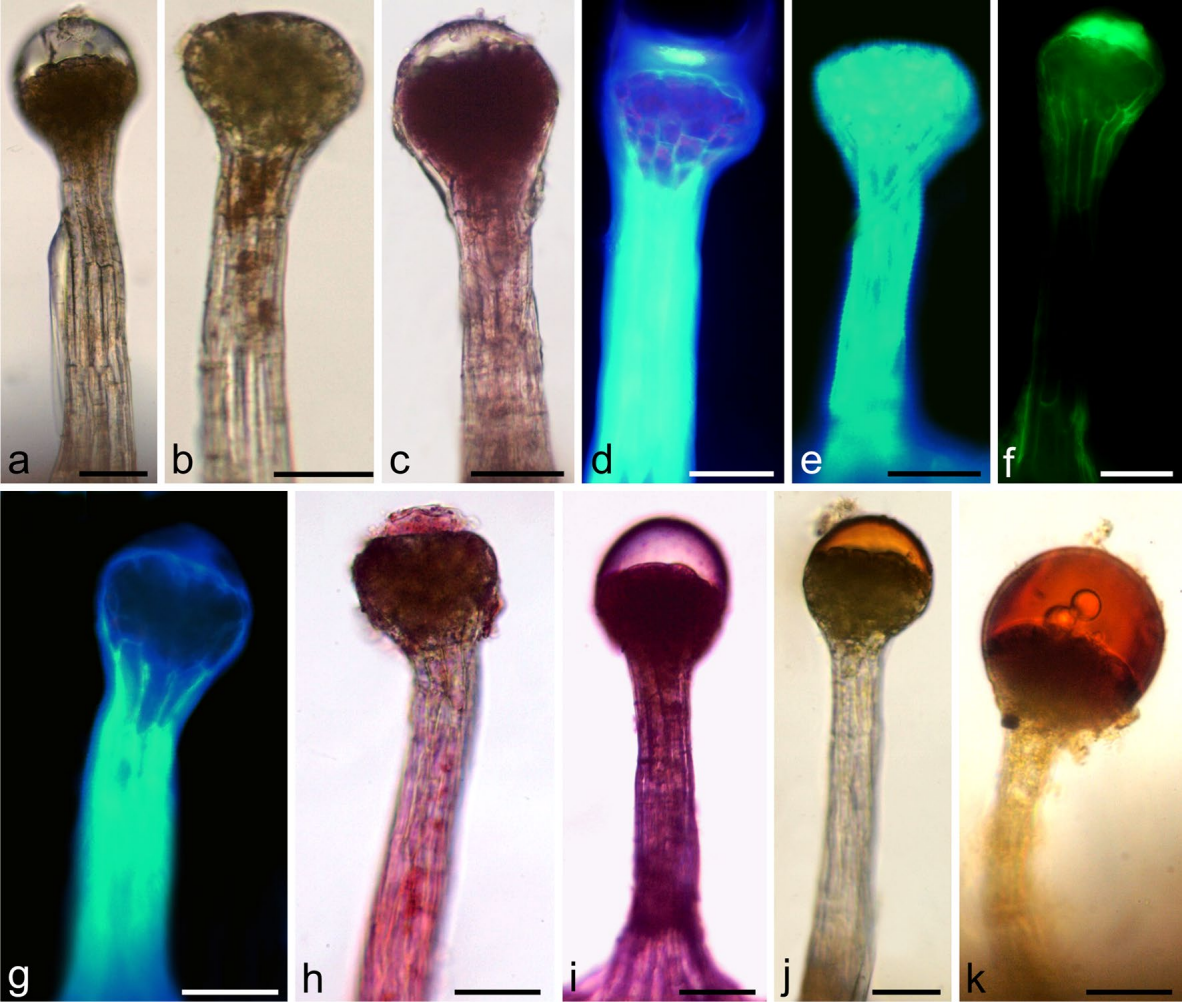
Histochemistry and fluorescence of glandular trichomes of Hartweg's locust – hydrophilic metabolites. **a–c, h–k**—LM, **d–g** – FM. **a** Staining of total polyphenols with ferric chloride. **b, c** Staining of tannins with dichromate potassium (**b**) and vanillin with hydrochloric acid (**c**). **d** Autofluorescence of phenolic acids and chlorophyll in the Cy5 filter set. **e–g** Fluorescence of flavonoids with aluminium chloride fluorochrome in the Cy5 (**e**) and TRITC (**f**) filter sets and with magnesium acetate fluorochrome in the Cy5 filter set (**g**). **h** Staining of acidic polysaccharides with Ruthenium Red. **i** Staining of neutral polysaccharides with periodic acid—Schiff's reagent (PAS). **j** Staining of proteins with an iodine iodide solution. **k** Staining of alkaloids with Wagner reagent. Scale bars = 100 µm (**a–k**)

Polyphenols, including tannins, were detected in the stalk and head cells (Fig. 5a–d). Polyphenols were stained brown by iron chloride (Fig. 5a), whereas potassium dichromate stained tannins light brown or brown (Fig. 5b). In turn, after application of vanillin in hydrochloric acid, tannins were stained burgundy (Fig. 5c), while phenolic acids present in the stalk and cuticle cells exhibited blue-light autofluorescence (Fig. 5d). In addition, white-yellow secondary fluorescence of flavonoids was visible in the presence of the magnesium acetate and aluminium chloride fluorochromes in the stalk cells, head cells, and secretion (Fig. 5e–g).

Acidic polysaccharides (mucilage, pectins) contained mainly in the stalk cells and trichome secretion were stained intensely purple in the Ruthenium Red reaction (Fig. 5h). In turn, neutral polysaccharides (cellulose) present in the head and stalk cells were stained cyclamen after treatment with Schiff’s reagent (PAS reaction) (Fig. 5i).

The reaction with the Lugol solution showed that the heads and secretion of the trichomes contained intensely yellow–orange-stained proteins (Fig. 5j). In turn, the reaction with Wagner's reagent confirmed the presence of dark orange-stained alkaloids in the trichome heads and secretion (Fig. 5k).

Additionally, red chlorophyll autofluorescence was observed in the cells of the trichome heads (Fig. 5d). There were no differences in the qualitative composition of secondary metabolites between the trichomes located on the examined organs (bracts, pedicels, and receptacles).

### Ultrastructure of immature and mature trichomes

**Fig. 6:**
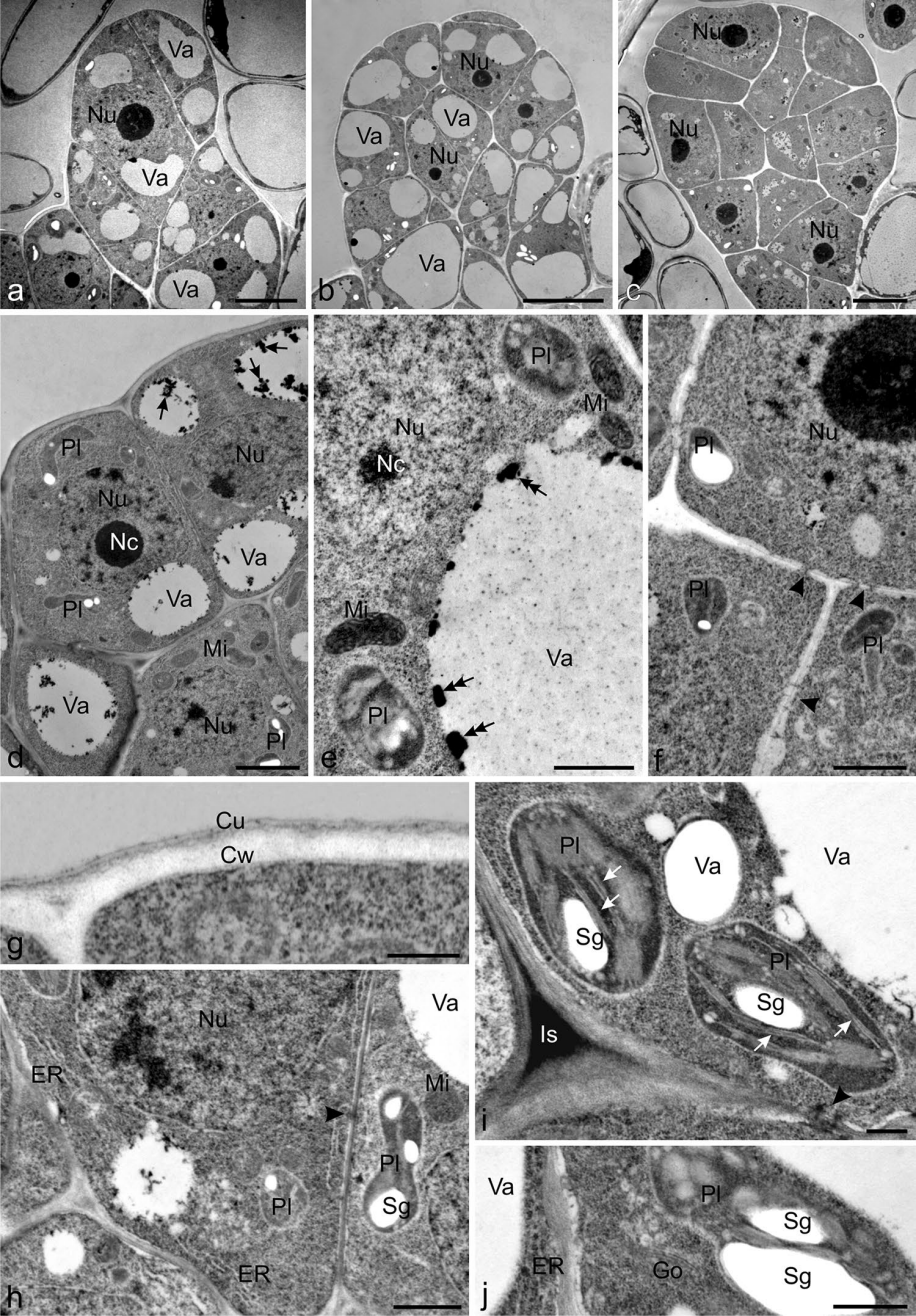
Ultrastructural traits of immature glandular trichomes of Hartweg's locust (TEM). **a–c** Trichomes in the initial stage of development. **d** Cells with a prominent nucleus with nucleoli, plastids, mitochondria, and small vacuoles with flocculent precipitates of tannins (black arrows). **e** Fragment of a cell with a vacuole with an electron-dense deposit of tannin near the tonoplast (double arrows) and cytoplasm with mitochondria and plastids. **f** Visible plastids in the cytoplasm and plasmodesmata (arrowheads) in the cell walls. **g** Apical fragment of a trichome with a thin cuticle on the surface. **h** Visible plastids with starch grains, mitochondria, ER profiles, and plasmodesmata (arrowhead) in the cell walls. **i** Visible plastids with starch grains and thylakoids (white arrows). **j** Visible plastids with starch grains, ER profiles, and Golgi apparatus with vesicles; Va vacuoles, Nu nucleus, Nc nucleoli, Pl plastids, Sg starch grains, Mi mitochondria, Pl plasmodesmata, Cw cell walls, Cu cuticle, ER endoplasmic reticulum, Is intercellular space, Go Golgi apparatus. Scale bars = 10 µm (**b**, **c**), 5 µm (**a**), 2 µm (**d**), 1 µm (**e, f, h**), 500 nm (**g**), 200 nm (**i, j**)

The cells of the immature trichomes in the initial stage of development were characterised by dense cytoplasm and the absence or presence of small vacuoles, sometimes containing electron-dense flocculent or, less often, solid tannin precipitates near the tonoplast (Fig. 6a–e). The cell walls of the trichomes exhibited the presence of plasmodesmata (Fig. 6f, h, i). A thin cuticle layer, which was approximately 2.5-fold thinner than the cell wall, covered the surface of the capitate protrusions forming immature trichomes (Fig. 6g). The cytoplasm contained a prominent nucleus with dark nuclei and dark areas exhibiting a substantial amount of heterochromatin (Fig. 6a–f), plastids with few thylakoids but most often with starch grains (Fig. 6d–f, h–j), mitochondria (Fig. 6d, e, h), parallel strands of endoplasmic reticulum (ER) profiles (Fig. 6h, j), and sparse Golgi apparatus with vesicles (Fig. 6j).

**Fig. 7:**
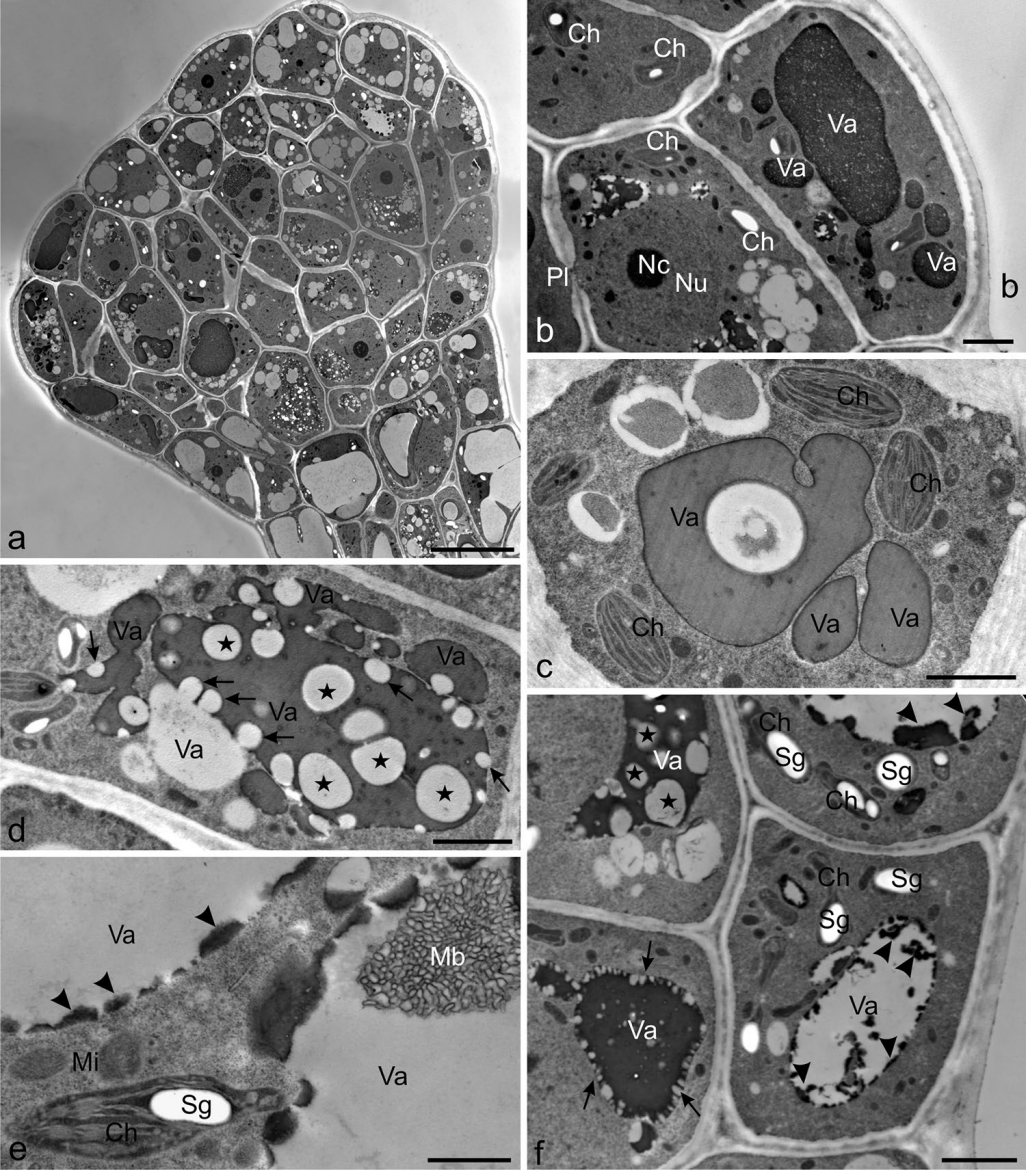
Ultrastructural traits of vacuoles in the secretory cells of mature glandular trichomes of Hartweg's locust (TEM). **a** General view of the secretory cells of a trichome head with a visible prominent nucleus and a varying degree of vacuolization. **b, c** Note the vacuoles with uniform and electron-dense content. Visible chloroplasts in the cytoplasm. **d, f** Visible vacuoles with numerous electron-transparent vesicles distributed in the dense electron content (stars) fused with vacuoles (arrows). Note the vacuoles merging with each other and forming a reticulate vacuolar system (**d**). **e, f** Visible dense electron deposits of condensed tannins (arrowheads) and membranous figures forming a characteristic pattern near the tonoplast (**e**); *Va* vacuoles, *Nu* nucleus, *Pl* plasmodesmata, *Ch* chloroplasts, *Sg* starch grains, *Mi* mitochondria, *Mf *membranous figure. Scal bars = 10 µm (**a**), 2 µm (**b-d, f**) 1 µm (**e**)

**Fig. 8:**
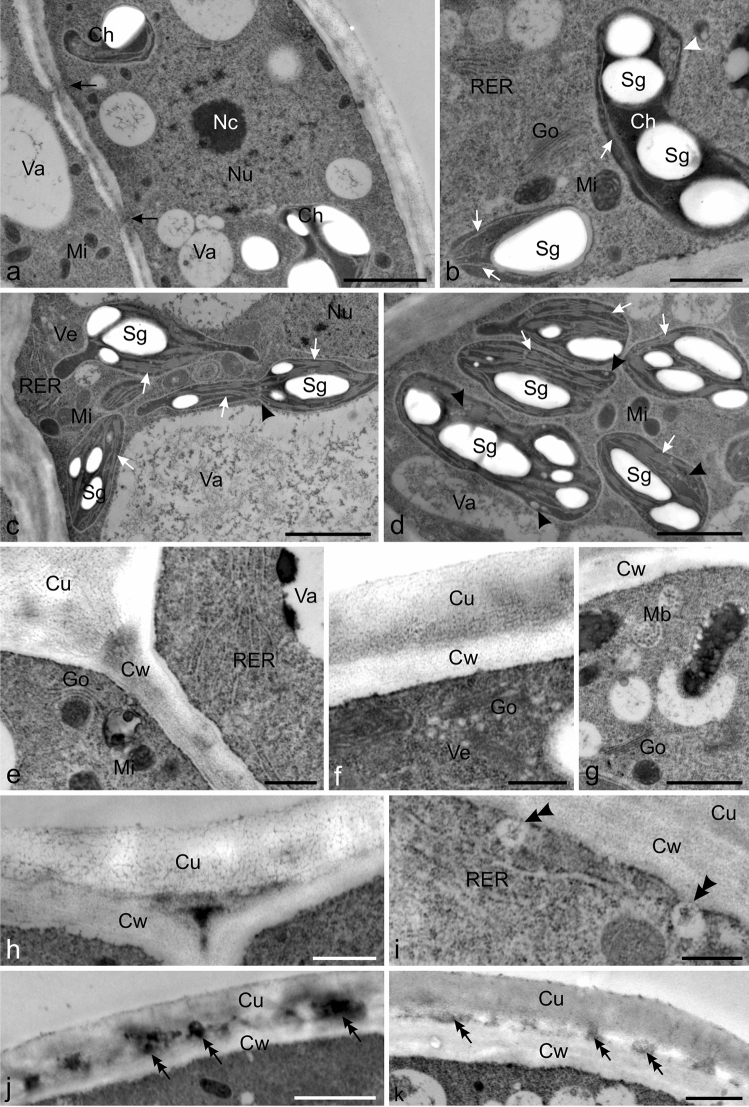
Ultrastructural traits of the secretory cells of mature glandular trichomes of Hartweg's locust (TEM). **a** A visible prominent nucleus with nucleoli, chloroplasts with starch grains, and small mitochondria in the cell of the peripheral part of the head. Note the plasmodesmata (black arrows) in the cell wall. **b** Visible chloroplasts with few thylakoids (white arrows), starch grains, and electron-transparent vesicles (arrowheads), mitochondria with well-developed mitochondrial cristae, rough ER profiles, and Golgi apparatus. **c, d** Visible mitochondria and polymorphic chloroplasts with numerous thylakoids (white arrows), large starch grains, and electron-transparent vesicles (arrowheads) in the cells of the central part of the head. Visible dense masses of fibrillar material in the vacuoles. **c** Note the clusters of RER profiles and electron-transparent vesicles near the plasmalemma. **e–k** Fragments of cells of the apical part of the head with a visible outer cell wall and cuticle. **e** Visible numerous RER profiles, mitochondria, and Golgi apparatus near the plasmalemma. **f** Visible Golgi apparatus with numerous vesicles near the plasmalemma. **g** Note the vesicular bodies and Golgi apparatus. **h** Visible reticulate cuticle with substantial thickness. **i** Visible RER profiles with vesicles fused with the plasmalemma (double arrowheads). **j, k** Note the initial phase of cuticle separation from the cell walls and the accumulation of electron-dense content in the emerging subcuticular space (double arrows); *Va* vacuoles, *Nu* nucleus, *Nc* nucleoli, *Ch* chloroplasts, *St* starch grains, *Mi* mitochondria, *Go* Golgi apparatus, *RER* rough endoplasmic reticulum, *Ve* vesicles, *Vb* vesicular bodies, *CW* cell wall, *Cu* cuticle. Scale bars = 2 µm (**a, c, k)**, 1 µm ( **b, d, g, j**), 500 nm (**e, f, h, i**)

The secretory cells in the heads of the mature fully developed glandular trichomes from the pedicels, bracts, and receptacles had a diverse structure (Fig. 7). Some cells had dense cytoplasm without vacuoles or with only a few small vacuoles. Other cells were highly vacuolated and usually had one large vacuole or numerous small vacuoles, sometimes merging with each other and forming a reticulate vacuolar system (Fig. 7a–d). Many vacuoles were filled with uniform electron-dense content (Fig. 7b, c), whereas the content in other cells had a complex structure (Fig. 7d, f), i.e. such vacuoles exhibited a specific “picture” of numerous electron-transparent different sized vesicles scattered in the electron-dense content or fused with the vacuoles (Fig. 7d, f). Dense masses of accumulated fibrillar material were visible in other vacuoles (Fig. 8c, d). Occasionally, electron-dense deposits of condensed tannins or membranous figures arranged in a characteristic pattern were present near the tonoplast inside the vacuoles (Fig. 7e, f).

The cytoplasm of the secretory cells of the head contained prominent cell nuclei with nucleoli (Figs. 7a,b, 8a), polymorphic chloroplasts (Figs. 7c–f, 8a–d), numerous mitochondria (Figs. 7e, 8b–e), rough ER (RER) profiles (Fig. 8b, c, e, i), and Golgi apparatus (Fig. 8a, b, d–f). In many plastids with electron-dense stroma, there were few thylakoids, large starch grains, and often numerous electron-transparent vesicles; other plastids contained higher numbers of thylakoids (Figs. 7c,f, 8a–d). Small sized mitochondria were often located near the chloroplasts and exhibited well-visible mitochondrial cristae (Figs.7e, 8b,e, i). The RER formed a cluster of well-developed profiles, often arranged concentrically, in the cells of the head apex (Fig. 8b, c, e, i). In their vicinity, there were electron-transparent vesicles, sometimes fused with the plasmalemma (Fig. 8c, i). Similar vesicles containing flocculent content were located in close proximity to the Golgi apparatus present near the outer walls of the apical head cells (Fig. 8f). This part of the trichomes sometimes exhibited the presence of vesicular bodies (Fig. 8g). The surface of the trichome heads was covered by a reticulate cuticle layer (Fig. 8e, f, h), which was usually substantially thicker than that in young trichomes and 1.5-fold thicker than the cell wall. TEM images of the apex of the heads of some trichomes showed the initial phase of cuticle detachment from the cell walls and accumulation of electron-dense content in the emerging subcuticular space (Fig. 8j–l). Numerous plasmodesmata were present in the cell walls between the head and stalk cells (Fig. 8a). Similar cellular structures were found mainly in the central part of the stalk cells (not shown). The trichomes from the bracts, receptacles, and pedicels had a similar ultrastructure.

## Discussion

The inflorescences and shoots of Hartweg's locust were covered by inconspicuous short non-glandular trichomes and massive glandular trichomes producing abundant sticky secretion. The trichomes present on the peduncles, pedicels, receptacles, bracts, and sepals had a similar structure, but their distribution, development stage, and length varied. The capitate trichomes in this species had distinct stalks. In contrast, the vegetative and generative organs of clammy locust (*R. viscosa* var. *viscosa*) bear sessile stalk-less glandular trichomes resembling papillae (Isely and Peabody^1984^; Peabody 1984). It has also been demonstrated that other *Robinia* species have only non-glandular trichomes: highly abundant in *R. hispida* or delicately tomentose in *R. pseudomexicana* and *R. pseudoacacia*. In turn, capitate glandular trichomes have been described so far only in *Coursetia* spp. from the tribe Robinieae (Lavin 1988), while various morphotypes of capitate glandular trichomes have been observed in other taxa from the subfamily Faboideae. Globose trichomes are present in representatives of the tribe Desmodieae (Freitas et al. 2014). Spherical and ellipsoid trichomes (Flores et al. 2019) and bulbous-based trichomes (Seixas et al. 2019; Vargas et al. 2019) have been described in plants from the tribe Phaseoleae, and cavitated secretory trichomes have been detected in members of the tribe Indigofereae (Marquiafável et al. 2009; Pignal and De Queiroz 2019). As suggested by these authors, the morphologically diverse trichomes in the different plant taxa in the subfamily Faboideae are important taxonomic traits useful for identification and classification of different taxa at the level of species, genus, subtribe, or tribe and for investigations of phylogenetic relationships.

The robust trichomes in Hartweg's locust were composed of a multicellular head and a massive multicellular and untypically multiseriate stalk. Although literature data show many morphotypes of capitate glandular trichomes in Faboideae representatives, partly similar trichomes to those present in Hartweg's locust have been described so far in *Ononis natrix* and *Parochetus communis* only by Gupta and Murty (1977) and Metcalfe and Chalk (1979). Our LM and TEM observations indicated that the peculiar trichomes in Hartweg's locust are merely a product of the epidermis of organs where they are located. Similar observations regarding the ontogenesis of trichomes in several representatives of the subfamily Caesalpinioideae (Fabaceae), which are morphologically similar to those in Hartweg’s locust, were presented by Souza et al. (2013). In contrast, in several *Caesalpinia* species from the same subfamily Caesalpinioideae, Rudall et al. (1994) and Lersten and Curtis (1996) showed the presence of external secretory structures with very similar morphology to that of the trichomes of Hartweg’s locust; however, they were produced by subepidermal tissues and protodermal cells. As suggested by Werker (2000) and de Barros et al. (2017b), trichomes and glands may have similar shapes and functions but different origin. The presence of similar trichome morphotypes in Hartweg’s locust (Faboideae) and in some members of Caesalpinioideae seems to indicate phylogenetic relatedness between these taxa.

The secretory trichomes in Hartweg's locust can be classified as persistent, as they were active both at the beginning and at the end of the vegetation period on different organs (unpublished data). Similar persistent secretory trichomes were reported by Matos and Paiva (2012) on young, mature, and ageing organs in members of the tribe Faboideae and by Souza et al. (2013) in members of the subfamily Caesalpinioideae. As shown by various authors, such persistent trichomes provide effective protection in the stage of juvenile leaves and inflorescences, i.e. a period of the greatest vulnerability to herbivore attacks, and in the rest of the vegetation period until fruiting (see Heil and McKey 2003; Paiva and Martins 2011).

The largest numbers of mature trichomes of Hartweg's locust underwent the secretory phase, during which secretion was accumulated in subcuticular spaces or spontaneously released through a pore in the cuticle on the trichome apex. Since Hartweg's locust trichomes are probably active throughout the growing season, we assume a repetitive secretion process. In agreement with our observations Oosthuizen and Coetzee (1983), confirm that accumulation and release of secretion are repeatable due to the development of a new cuticle beneath the ruptured layer. Similar accumulation of secretion in the subcuticular space and its release through a cuticle perforation has been reported in other representatives of the subfamily Faboideae (Zarinkamar and Sharebian 2016; Vargas et al. 2019) and in the subfamily Caesalpinioideae (Paiva and Machado 2006; Souza et al. 2013).

The trichomes and secretion of Hartweg's locust contained two types of metabolites, i.e. hydrophilic and lipophilic compounds (Table 1). Similar groups of secondary metabolites have been identified in trichome secretions of various Faboideae and Caesalpinioideae members (e.g. Palermo et al 2017; Silva et al. 2017). In the present study, we did not observe differences in the composition of the trichome secretion or in the ultrastructure of trichomes collected from the different parts of inflorescences, which suggests that these trichomes served similar functions. Similarly, Marquiafável et al. (2009) suggest that morphologically varying secretory trichomes may not differ in the chemical composition of their secretion and/or their role in the plant.

The secretion in the analysed trichomes was released by secretory cells through the plasmatic membranes and outer walls of the apical trichome cells via granulocrine and/or eccrine modes of secretion. The presence of vesicles fused with the plasmalemma, the proximity of dictyosomes and ER producing these vesicles, and the multivesicular bodies visible in these areas indicate the granulocrine mode of secretion. The mechanism of granulocrine secretion has also been described in various members of Fabaceae (e.g. Healy et al. 2009; Vargas et al. 2019). The authors have shown that secretion-filled vesicles detaching from ER and dictyosomes fuse with the plasmalemma releasing their content via exocytosis. The presence of numerous mitochondria and chloroplasts in the Hartweg's locust stalk and head cells may prove the parallel eccrine secretion process, i.e. active transport of secretion components or diffusion directly through the plasma membrane. As suggested by various authors, the highly organised cytoplasm, large nuclei, and numerous chloroplasts and mitochondria are typical traits of secretory tissues with high metabolic activity (Machado et al. 2006; Konarska 2020). Many researchers confirm that two alternative granulocrine and eccrine mechanisms of secretion can operate simultaneously in the case of various derivatives that are not synthesised in the same organelles and in the same cell compartments (Rodrigues and Machado 2012; Sá‐Haiad et al. 2015).

The chloroplasts with red autofluorescence in the cells of Hartweg's locust trichomes had distinct thylakoids and starch grains. The presence of chloroplasts with starch grains in the cells of various exogenous secretory structures has been detected in other members of Fabaceae (Matos and Paiva 2012; Meira et al. 2014). The authors report that secretory cells containing chloroplasts can carry out photosynthesis to provide precursors and energy for the production of secreted substances via degradation of carbohydrates. Various authors claim that plastids in trichomes also play a role in the biosynthesis, accumulation, and secretion of polysaccharides (Paiva and Machado 2008) and alkaloids (Wink and Roberts 1998). These metabolites were present in the secretory cells of the analysed *Robinia* taxon as well.

The glandular trichomes of Hartweg's locust underwent the initial secretory phase in the stage of the synthesis of polysaccharides, proteins, or tannins, as shown by TEM. Many authors claim that the synthesis of various metabolites is not synchronous. In young mature trichomes, hydrophilic compounds are produced first, whereas hydrophobic compounds such as fats, essential oils, or terpenic resins are produced later (Muravnik et al. 2016; Silva et al. 2017). The presence of numerous RER profiles and Golgi apparatus in the glandular trichomes of Hartweg's locust suggests a role of these structures in the synthesis and transport of proteins and polysaccharides. Similarly, research results reported by other scientists documented the role of RER and Golgi apparatus in the biosynthesis of carbohydrate and protein secretory products (Matos and Paiva 2012; Guo and Zhou 2019). Moreover, RER profiles are involved in the production of phenolic compounds (Marinho et al. (2015), whose presence was also detected in the secretory cells of Hartweg's locust trichomes.

The histochemical assays and LM and TEM observations showed contents of soluble and condensed tannins in cell vacuoles in the mature trichomes in Hartweg's locust. Vacuoles filled with soluble tannins frequently contained transparent vesicles forming a specific pattern against the electron-dense content. These vesicles presumably deposited metabolites formed in the cytoplasm other than tannins in the vacuoles, as shown by the histochemistry and fluorescence assays. Such microvacuoles merging with larger vacuoles where secretory products were temporarily accumulated were also observed by other researchers in various secretory structures (Happel et al. 2004; Paiva 2016). These microvacuoles were filled with polysaccharides (Fahn and Shimony 1996), flavonoids (Heinrich et al. 2002), or alkaloids (Wink and Roberts 1998). As demonstrated by many authors, toxic tannins can form complexes with proteins, alkaloids, mucilage, pectins, carbohydrates, and fats (Hagerman 2012; Ramaswamy et al. 2013; Zhao et al. 2013; Jakobek 2015). As suggested by Klein and Roos (2009), to minimise the risk of self-intoxication with such toxic metabolites as phenolic compounds/tannins and alkaloids, plants target these metabolites into vacuolar compartments with low metabolic activity and neutralise them by formation of various safe complexes. In turn, some soluble tannins may have high oxidative capacity, and their toxic oxidation products significantly reduce the growth and development of insect herbivores (Barbehenn et al. 2006; Salminen and Karonen 2011). The presence of tannins is characteristic for different *Robinia* species and some representatives of the tribe Robiniea (Kumar and Horigome 1986; Lavin 1988) and is a relatively common feature of the entire family Fabaceae (Barros et al. 2017a; Marinho et al. 2018). Also other groups of phenolic compounds, e.g. flavonoids and phenolic acids, were detected in the secretion and secretory cells of the Hartweg's locust trichomes. They protect plants against bacterial or viral attack by counteracting excessive production of reactive oxygen species (Khalid et al. 2019) and via their antifungal and antifeedant activities (Bergau et al. 2015; Kundan et al. 2019). An additional function of flavonoids is to protect plants against UV-B radiation (Tang et al. 2020) and regulate auxin transport (Wood 2017). The presence of robinin, i.e. a flavonoid with therapeutic potential, in *R. viscosa* organs has been confirmed by Maksyutina (1969).

Proteins and alkaloids were detected in the head cells and secretion in the Hartweg's locust trichomes. Proteins in secretory tissues can bind such hydrophobic ligands as flavonoids and plant hormones (Fernandes et al. 2008), serve as flavonoid-type transporters (Hjernø et al. 2006), and provide additional protection against fungi and parasites (Mayer et al. 2011). The presence of alkaloids confirms the toxic effects of this plant. The organs of various *Robinia* species also contain toxic lectins (Loris et al. 1998; McPherson 2010). Many studies have demonstrated that lectins from different plants, including *R. pseudoacacia*, can be applied in bioscience and biomedicine as constituents of anti-cancer drugs. They are also intestinal, metabolic, and hormonal regulators, immune reagents, and probiotic/prebiotic oral supplements (see Barre et al. 2002; Pusztai et al. 2008 and references wherein). Alkaloids are regarded as the most effective insecticides and repellents (Jing et al 2014; Babaousmail et al. 2018). In Fabaceae representatives, alkaloids have been shown to have biological and ecological significance and potential to be taxonomical markers (Aniszewski 2015). They were detected in trichomes located on all the examined floral parts in Hartweg's locust, except for floral corollas, which do not bear secretory trichomes. We suppose that the corollas of Hartweg's locust may not have toxic properties, likewise *R. pseudoacacia* flowers, which are edible and can be used as important herbal raw material (Rosu et al. 2012; Chen and Dai 2014). We believe that further investigations are necessary to determine the quantitative and qualitative content of alkaloids in Hartweg's locust corollas and other organs and estimate the real degree of toxicity of this taxon. As suggested by various authors, the distribution of alkaloids is specific to plant taxa. There may be large differences in the alkaloid content between plants of one family but similarities between species of the same genus (Jing et al. 2014; Lokhande and Pathak 2018).

The abundant secretion of the analysed trichomes was highly viscous and formed an adhesive layer on the surface of the examined organs, to which arthropods and aeroplankton elements adhered. The extreme adhesiveness of the secretion was associated with its content of fats and mucilage, as confirmed by the histochemical assays. The viscosity of the secretion hindered foraging and movement of herbivores on the plant surface and may have limited the development of fungal and bacterial pathogens (Weinhold and Baldwin 2011; Munien et al. 2015). Moreover, as suggested by Oliveira et al. (2017), the mucilaginous secretion may constitute a specific adaptation to high levels of light irradiation, high temperatures, and low water availability. Highly adhesive secretion has also been detected in trichomes of representatives of other Faboideae and Caesalpinioideae subfamilies (Southwood 1986; Silva et al. 2017).

In Hartweg's locust, the viscosity of the secretion provided by mucilage and fats and the content of flavonoids, proteins, and alkaloids constitute the first very strong protective barrier against pathogens and smaller pests. To fight stronger herbivorous arthropods overcoming the viscosity and toxicity of secretions, the plant has created another barrier, i.e. toxic tannins accumulated in trichome cells and other alkaloid-reinforced polyphenols. Peter et al. (1995) and Lima et al. (2017) have identified three types of action of Fabaceae trichomes as an insect-resistance mechanism: (i) a physical barrier limiting the contact between the plant and insects, (ii) production of toxic metabolites poisoning insects upon contact with the secretion, and (iii) production of viscous secretory compounds on the plant surface to immobilise insects. As indicated by our observations, all these mechanisms of triple protection of plants against pests operate in Hartweg's locust*.*

## Conclusions

The original results obtained in this study indicate that the glandular trichomes present on the flowers of Hartweg's locust belong to persistent and repetitive secretion process trichomes. Capitate trichomes located on different parts of the inflorescence differ in the distribution, development stage, and length, but have the same microstructure and undoubtedly serve the same protective function. Their heterogeneous secretion contains mucilage, fats, flavonoids, proteins, and alkaloids and can thus be an efficient defence against herbivores and pathogens. Secretion is transported via the symplast pathway and is collected in the subcuticular space from where it is released through cuticle pores. The simultaneous functioning of two mechanisms of secretion, i.e. granulocrine and eccrine, suggests that different derivatives are synthesised in the different organelles and cell compartments. Our study, which is the first report on the type of exudates and the morphology, anatomy, and ultrastructure of the glandular trichomes in Hartweg's locust flowers, has implications for the taxonomical classification of the tribe Robinieae, as they are important diagnostic traits used in taxonomy and systematics of plants. These novel findings confirm the potential of Hartweg's locust to be a source of new bioactive compounds.

### *Author contributions statement*

AK, BŁ conceptualization, AK, BŁ methodology, AK, BŁ formal analysis and investigation, AK writing the manuscript, AK, BŁ photographs. All authors read and approved the manuscript.
